# Deciphering spatially distinct immune microenvironments in glioblastoma using ferumoxytol and gadolinium-enhanced and FLAIR hyperintense MRI phenotypes

**DOI:** 10.1093/noajnl/vdad148

**Published:** 2023-11-08

**Authors:** Jared Stoller, Cymon N Kersch, Leslie L Muldoon, Prakash Ambady, Christina A Harrington, Rongwei Fu, Ahmed M Raslan, Aclan Dogan, Edward A Neuwelt, Ramon F Barajas

**Affiliations:** Department of Diagnostic Radiology, Oregon Health and Science University, Portland, Oregon, USA; Department of Radiation Medicine, Oregon Health and Science University, Portland, Oregon, USA; Department of Neurology, Oregon Health and Science University, Portland, Oregon, USA; Department of Oncology, Providence Hospital, Portland, Oregon, USA; Department of Molecular and Medical Genetics, Oregon Health and Science University, Portland, Oregon, USA; Knight Cancer Institute, Oregon Health and Science University, Portland, Oregon, USA; School of Public Health, Oregon Health & Science University-Portland State University, Portland, Oregon, USA; Department of Neurosurgery, Oregon Health and Science University, Portland, Oregon, USA; Department of Neurosurgery, Oregon Health and Science University, Portland, Oregon, USA; Department of Neurology, Oregon Health and Science University, Portland, Oregon, USA; Department of Neurosurgery, Oregon Health and Science University, Portland, Oregon, USA; Department of Veterans Affairs Medical Center, Portland, Oregon, USA; Department of Diagnostic Radiology, Oregon Health and Science University, Portland, Oregon, USA; Knight Cancer Institute, Oregon Health and Science University, Portland, Oregon, USA; Advanced Imaging Research Center, Oregon Health and Science University, Portland, Oregon, USA

**Keywords:** ferumoxytol, glioblastoma, MRI, transcriptomics

## Abstract

**Background:**

MRI with gadolinium (Gd)-contrast agents is used to assess glioblastoma treatment response but does not specifically reveal heterogeneous biology or immune microenvironmental composition. Ferumoxytol (Fe) contrast is an iron nanoparticle that localizes glioblastoma macrophages and microglia. Therefore, we hypothesized that the use of Fe contrast improves upon standard Gd-based T1-weighted and T2/FLAIR analysis by specifically delineating immune processes.

**Methods:**

In this, HIPAA-compliant institutional review board-approved prospective study, stereotactic biopsy samples were acquired from patients with treatment-naïve and recurrent glioblastoma based on MR imaging phenotypes; Gd and Fe T1 enhancement (Gd+, Fe+) or not (Gd–, Fe–), as well as T2-Flair hyperintensity (FLAIR+, FLAIR–). Analysis of genetic expression was performed with RNA microarrays. Imaging and genomic expression patterns were compared using false discovery rate statistics.

**Results:**

MR imaging phenotypes defined a variety of immune pathways and Hallmark gene sets. Gene set enrichment analysis demonstrated that Gd+, Fe+, and FLAIR+ features were individually correlated with the same 7 immune process gene sets. Fe+ tissue showed the greatest degree of immune Hallmark gene sets compared to Gd+ or Flair+ tissues and had statistically elevated M2 polarized macrophages, among others. Importantly, the FLAIR+ Gd+ and Fe– imaging phenotypes did not demonstrate expression of immune Hallmark gene sets.

**Conclusions:**

Our study demonstrates the potential of Fe and Gd-enhanced MRI phenotypes to reveal spatially distinct immune processes within glioblastoma. Fe improves upon the standard of care Gd enhancement by specifically localizing glioblastoma-associated inflammatory processes, providing valuable insights into tumor biology.

**Key Points**

- Glioblastoma immune heterogeneity can be defined by MRI phenotypes.
- Fe+ demonstrates the highest degree of differentially expressed immune gene signatures.
- Higher levels of innate immune cellular infiltration are observed within Fe+ samples.

**Importance of the Study**

Insight into the biological processes responsible for MRI phenotype formation is critical to assessing response to therapy. This prospective study demonstrates that the heterogeneous glioblastoma immune microenvironment can be assessed by MRI where differences in gadolinium and ferumoxytol iron nanoparticle contrast enhancement provide phenotypes associated with distinct immune-related transcriptomic signatures.

Glioblastoma is the most common primary brain malignancy in adults. Standard of care therapy, maximal safe resection followed by temozolomide-based chemoradiation, results in a median survival of 14.6 months.^1^ The inability to achieve meaningful long-term survival is multifactorial but in part stems from an incomplete understanding of the tumor immune microenvironment.

Therapeutic resistance results from a variety of mechanisms including tumor immune evasion potentiated by a hypoxic immune microenvironment predominately comprised of pro-tumoral polarized M2 macrophages, among other cellular immune constituants.^2,3^ As such, it is ­critically important to better understand the heterogeneity of the tumor immune microenvironment. The ability to define the heterogeneity of the immune microenvironment has the potential to improve therapeutic approaches to better incorporate targeted immunotherapies.^4^

Gadolinium (Gd)-enhanced MRI is routinely utilized to monitor treatment response but, is incapable of providing biologically specific information about treatment outcomes or the tumor immune microenvironment. The emerging field of imaging genomics is exploring the possibility of utilizing imaging biomarkers to define genetic expression patterns with quantitative metrics derived from imaging data.^5–7^ Initial studies suggest that multivariate imaging analysis of Gd enhancement, T2/FLAIR signal hyperintensity, diffusion-weighted imaging, and relative cerebral blood volume may identify distinct immune microenvironments.^8^

Ferumoxytol (Fe) is an FDA-approved iron-based nanoparticle with ferromagnetic properties used “off label” as an MRI contrast. Fe-enhanced MRI has the ability to define post therapeutic neuroinflammation regions, potentially indicating the presence of tumor-associated macrophages (TAMs) and specific immune niches.^9,10^ The proposed mechanism for T1-weighted enhancement is through tumor-associated macrophage (TAM) phagocytosis of Fe.^11^ This suggests Fe may be an alternative approach for the biologically specific identification of TAMs in the glioblastoma immune microenvironment and is likely to lend insights into specific immune mechanisms, and its copresence with Gd enhancement or other MRI features may further help distinguish unique immune niches.^12^ Therefore, we hypothesized that the use of Fe contrast improves upon standard Gd-based T1-weighted and T2/FLAIR analysis by specifically delineating the heterogeneous immune microenvironment.

In this prospective study, we aimed to evaluate the clinical utility of Fe enhancement in delineating immune genomic signatures and cellular constituents within glioblastoma. Our results demonstrate that Fe enhancement uniquely identifies differentially expressed immune signatures, surpassing the capabilities of Gd. Complementing Gd, Fe-enhanced MRI provides spatially distinct information about various innate immune cell populations within the tumor immune microenvironment. This advancement in medical knowledge using MRI phenotypes has significant clinical implications if validated in a larger cohort, providing valuable insights into immune processes related to therapy resistance and potentially offering a better tool for understanding the heterogeneity of immune responses in glioblastoma.

## Materials and Methods

### Patient Cohort

Adult patients referred to the Oregon Health and Science University (OHSU) Neurological Surgery Service for resection of clinically suspected glioblastoma were prospectively enrolled in this Health Insurance Portability and Accountability Act–compliant, institutional review board-approved study between February 2018 and April 2019. The institutional review process independently assessed all required levels of medical, ethical, and safety considerations prior to study approval. Patients younger than 18 years of age or with a medical history of systemic primary cancer were not enrolled in this study. Three patients with suspected glioblastoma were excluded from analysis because of Isocitrate Dehydrogenase (IDH) mutational status, histological diagnosis of Grade 2 Astrocytoma, or histological diagnosis of B-cell Lymphoma.

### MR Imaging Protocol

All patients underwent preoperative 3T MRI (Ingenia; Philips Healthcare) on 2 consecutive days. A 3D T1 TFE (TR = 1330 ms, TE = 4.84 ms, 250 mm^2^ FOV, 256 mm^2^ matrix, 1.25 mm slice thickness) was performed before, 10 min after the i.v., administration of 0.2 mmol/kg Dotarem-contrast (gadoterate meglumine, Gd), and 24 h after i.v., administration of Fe administration. Fe contrast was infused over 15 min at a dose of 7 mg/kg up to 510 mg diluted to a final volume of 34 mL in normal saline.^10^ FLAIR (TR = 9000 ms, TE = 102 ms, 220 mm^2^ FOV, 512 mm^2^ matrix, 5 mm slice thickness, IR = 180°) imaging was performed prior to contrast administration.

### MRI and Tissue Analysis

T1 and FLAIR weighted imaging was integrated into an intraoperative neuronavigational device for tissue sampling based on signal intensity (T1 Gd-enhancing [Gd+] or not [Gd–], T1 Fe-enhancing [Fe+] or not [Fe–], and FLAIR hyperintensity [FLAIR+] or not [FLAIR–]). The presence of T1 enhancement was defined by an attending neuroradiologist (R.F.B. certified by the American Board of Radiology with a certificate-added qualification in neuroradiology with more than 16 years of experience) based on signal intensity of normal-appearing white matter. Regions within each tumor were noted to have any combination of the 3 imaging features and were then stereotactically located at the time of resection to obtain tissue with the known imaging characteristics. All tissue specimens were collected with open image-guided stereotactic biopsy techniques by a neurological surgeon (A.R., A.D.; with more than 20 years of experience). Screenshots and MR imaging coordinates of sampling sites were recorded to confirm the accuracy of preplanned locations. Tissue specimens were immediately placed in RNAlater solution for 12 h and then stored at –80°C until RNA microarray analysis.

Total RNA extraction from all tissue samples using Quick-DNA/RNA Miniprep kits (Zymo Research) was performed by the OHSU Gene Profiling Shared Resource. Five nanograms of total RNA from each sample were amplified and biotin-labeled using the GeneChip™ 3ʹ IVT Pico Kit (Applied Biosystems) as per manufacturer recommendations. Labeled targets were hybridized with GeneChip™ Clariom S Human Arrays (Thermo Fisher Scientific) and arrays were scanned using the GeneChip Scanner 3000 with a 7G upgrade. GeneChip image processing was performed with GeneChip Command Console software v. 3.1.1 and probe set summarization and CHP file generation were performed using Transcriptome Analysis Console v 4.0.2.15 (Thermo Fisher Scientific).

### Gene Set Enrichment Analysis (GSEA) and Cytoscape Visualization

Gene Set Enrichment Analysis (GSEA software V4.2.3) assessed differential transcriptomic expression between MRI phenotypes.^13^ Molecular signature database (MsigDB), Gene Ontology (C5), or Hallmark (H) sets provided reference cancer-associated biological processes.^13^ Significantly enriched gene sets demonstrated a normalized enrichment score (NES) equal to or greater than 2.0 and a false discovery rate *q*-value less than 0.05, allowing for stringent correction of multiple comparisons. Highly enriched gene sets had the highest degree of statistically significant differential expression denoted by NES greater than 2.5. Cytoscape (V3.10.0)^14^ clustering map nodes (denoting at least 1 gene set) required at least 7 connected nodes and a *P*-value cutoff of .05 to be visualized.

### Cibersortx

Cibersortx^15^ was utilized to estimate the cellular immune composition of each tissue sample using predefined gene expression values.^16^ Gene expression data was uploaded directly to the Cibersortx online portal for processing. The statistically significant differential cellular expression between MRI phenotypes was assessed by a *t*-test with a *P*-value of less than 0.05.

### Heatmaps

The pheatmap package within R (version 4.3.0) was used to compute heatmaps.^17^ Heatmaps visualize unsupervised hierarchical clustering of predefined gene sets and sample characteristics. Clustering was performed with Ward’s method with the Euclidean distance metric. Gene expression displayed was first log_2_ transformed and then *z*-score normalized from –3 to 3 based on deviation from mean expression.

## Results

### Patient Population

In total, 10 adult patients with suspected IDH wild-type disease were enrolled after written informed consent. However, only 7 patients were found to have IDH wild-type disease (mean age, 61.75 years ± 13.66: age range, 44–77 years) 6 men (mean age, 63 years ± 14.25; age range, 44–77 years) and 1 woman (age 53 years) (Supplemental Table 1). For the 7 IDH wild-type patients, a total of 62 biopsy specimens were obtained and assessed; Fe+, *N* = 40; Fe–, *N* = 22; Gd+, *N* = 36; Gd–, *N* = 26) (Supplemental Table 1). Two patients provided tissue samples at the time of initial diagnosis prior to Stupp protocol chemoradiotherapy (CRT). The remaining tissue samples were obtained at the time of failed CRT.

### Fe MRI Phenotype Denotes Immune Processes

**Figure 1.**
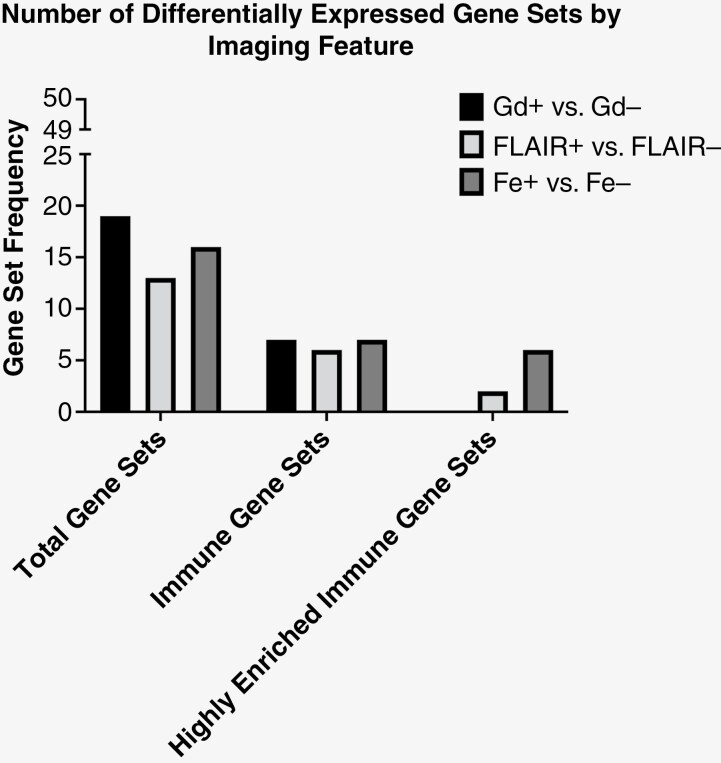
Differential gene expression by MRI phenotype. Differing MRI phenotypes demonstrated various degrees of differential gene expression. For all possible gene sets (*N* = 50), Gd was found to have the greatest degree of differential expression (*N* = 19). However, FLAIR (*N* = 13) and Fe (*N* = 16) also showed similar findings. When analysis is confined to only knowing immune-related processes (middle, immune gene sets). We observed similar differential expression between all of the 3 MRI phenotypes. However, when differential expression of these immune gene sets is further stringently controlled (right, highly enriched) with an NES of 2.5, we observed the Fe MRI phenotype to have the highest number of highly differentially expressed immune gene sets (*N* = 6). Conversely, no highly enriched immune gene sets were observed to be differentially expressed between Gd+ and Gd– tissues. A detailed list of the gene sets is found in Tables 1 and 2, and Supplemental Table 3

GSEA analysis allowed for the assessment of differential gene expression between tissue samples. This demonstrated that both Gd+ and Fe+ tissue samples were correlated with the same 7 immune process gene sets; allograft rejection, blood coagulation cascade, complement cascade, IL6 STAT3 signaling during acute phase response, inflammation, interferon alpha response, and interferon gamma response (Table 1). FLAIR+ tissue samples had nearly identical results to Gd+ and Fe+ imaging features with the absence of the complement cascade gene set. This suggested the presence of enhancement and FLAIR hyperintensity was positively associated with elevated immune process gene set expression. Given the degree of differential gene expression, we assessed a more stringent NES score of 2.5 for highly enriched immune gene sets within tissue samples. With a NES score of 2.5, we observed only FLAIR+ (*N* = 2 gene sets) and Fe+ (*N* = 6 gene sets) tissue samples demonstrated differential expression of highly enriched immune process gene sets (Figure 1 and Table 1). To assess for differential immune gene set expression by type of MRI contrast we confined the analysis to FLAIR+ tissues for additional GSEA hallmark gene set analysis. This demonstrated both Gd+ and Fe+ regions had similar expression of differential immune process gene sets. A multiparametric approach was then utilized to further explore the contrast imaging feature most responsible for the observed differential gene set enrichment. Fe+ Gd– phenotype demonstrated differential expression of the same 7 immune process gene sets. However, the Gd+ Fe– phenotype did not demonstrate any differential expression of immune process gene sets suggesting that the observed immune process may be substantially explained by Fe enhancement (**Table 2**).

**Table 1.** Differential Immune Gene Sets Between MRI Phenotypes for All Tissues

Gd+ vs. Gd–	FLAIR+ vs. FLAIR–	Fe+ vs. Fe–
Interferon-gamma response	Interferon-gamma response^*^	Interferon-gamma response^*^
Interferon-alpha response	Interferon-alpha response^*^	Interferon-alpha response^*^
IL6 STAT3 signaling during acute phase response	IL6 STAT3 signaling during acute phase response	Allograft rejection^*^
Blood coagulation cascade	Inflammation	IL6 STAT3 signaling during acute phase response
Inflammation	Allograft rejection	Inflammation^*^
Complement cascade	Blood coagulation cascade	Complement cascade^*^
Allograft rejection		Blood coagulation cascade

^*^ Highly enriched gene set with NES of at least 2.5. Note: Gene sets are listed in decreasing NES.

**Table 2.** Differential Immune Gene Sets Between Enhancement in FLAIR+ Tissues

Gd+ vs. Gd–	Fe+ vs. Fe–	Gd+ vs. Fe–	Gd– vs. Fe+
Interferon-gamma response^*^	Interferon-gamma response^*^	None	Interferon-gamma response^*^
Interferon-alpha response^*^	Interferon-alpha response^*^		Interferon-alpha response^*^
Allograft rejection^*^	Allograft rejection^*^		Allograft rejection^*^
Inflammation^*^	Inflammation^*^		Inflammation^*^
IL6 STAT3 signaling during acute phase response^*^	IL6 STAT3 signaling during acute phase response^*^		IL6 STAT3 signaling during acute phase response
Complement cascade^*^	Complement cascade		Complement cascade
Blood coagulation cascade	Blood coagulation cascade		Blood coagulation cascade

^*^ Highly enriched gene set with NES of at least 2.5. Note: Gene sets are listed in decreasing NES.

### Relationship of MRI Phenotypes to Interrelatedness of Differential Gene Expression

**Figure 2.**
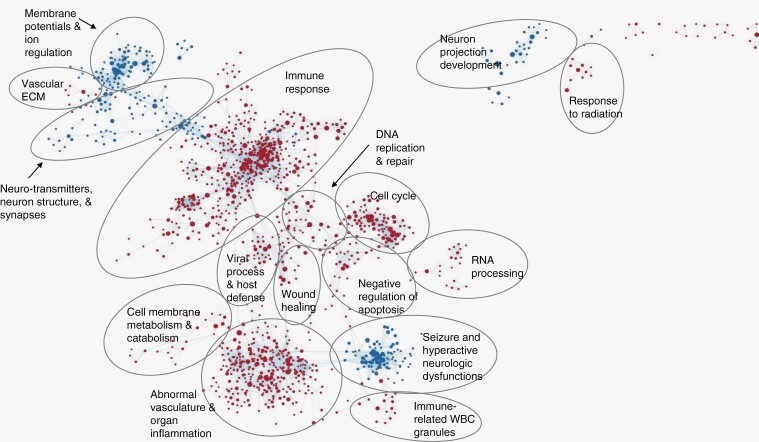
Fe MRI phenotype is associated with numerous immune biological processes. in these cytoscape maps, each dot is a node comprised of at least 1 gene set, each line connecting the nodes is called an edge and denotes interrelatedness. Red nodes are gene sets that are positively correlated, and blue nodes are gene sets that are negatively correlated with the Fe+ feature. Numerous gene signatures are observed to be interrelated with immune response and abnormal vasculature, regulation of apoptosis, and inflammation being the largest biological processes found to be positively associated with the Fe MRI phenotype.

The interrelatedness of genomic expression was visualized using Cytoscape maps. This open-source platform arranges the gene sets based on relative expression allowing for visualization of molecular interaction networks and biological pathways. The Fe-enhancing feature was correlated with upregulation of an immune system response, abnormal vasculature, inflammation, and negative regulation of apoptosis as well as a downregulation of membrane potential and ion regulation and neuron projection development (Figure 2). The Gd-enhancing feature was correlated with an upregulation of an immune response with a strong humoral component in the tumor microenvironment (Figure 2). It was also correlated with the downregulation of neuron development membrane potential and ion regulation (Supplemental Figure 1). The FLAIR hyperintensity was correlated with an upregulation of both positive and negative immune regulatory pathways as well as DNA damage and repair genes and a downregulation with membrane potential, ion exchange, and neurotransmitter transport regulation (Supplemental Figure 2).

### Differential Immune Cell Expression and Inflammatory Processes by MRI Features

**Figure 3.**
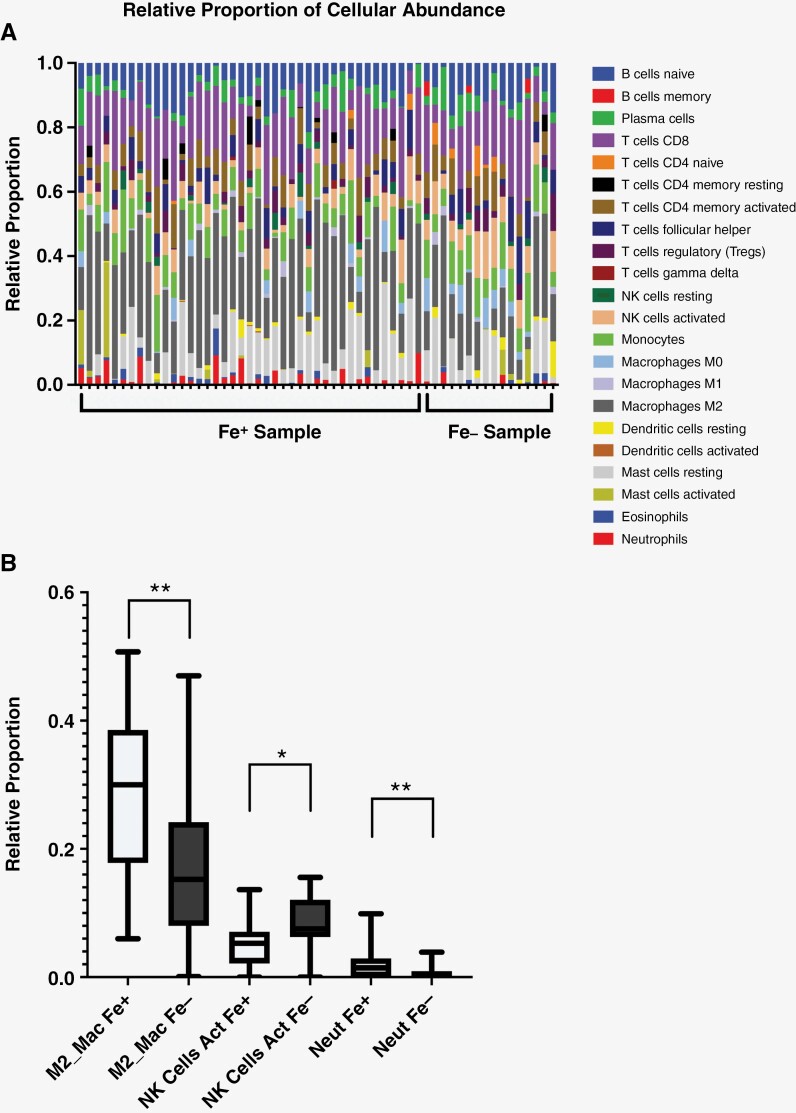
Immune cellular composition of Fe MRI phenotype. (A) Cibersortx analysis for of the Fe MRI phenotype demonstrates a large variation in differential expression of immune cells between the Fe+ and Fe– negative tissues. (B) Only innate immune cells were found to be significantly different by Fe MRI feature. This included M2 macrophages, activated natural killer cells, and neutrophils. Note the degree of significance (^*^P < .05, ^**^P < .005)

Cibersortx software determines the cellular makeup through the deconvolution of microarray data. Fe-enhancing regions demonstrated differential innate immune cellular composition (Figure 3A) with significantly elevated levels of M2-polarized macrophages and neutrophils (Figure 3B). Fe-nonenhancing regions demonstrated significantly elevated levels of activated natural killer cells. The FLAIR hyperintense and to a lesser degree Gd-enhancing regions also demonstrated differential immune cellular composition (Supplemental Figures 3 and 4). FLAIR hyperintense regions demonstrated a cellular immune composition consistent with a pro-inflammatory response comprised of resting memory CD4 T cells, T-cell CD4 memory resting and activated, M1 macrophages, and eosinophils suggesting that tumor tissue, experienced more inflammation than healthy brain tissue.^3^

**Figure 4.**
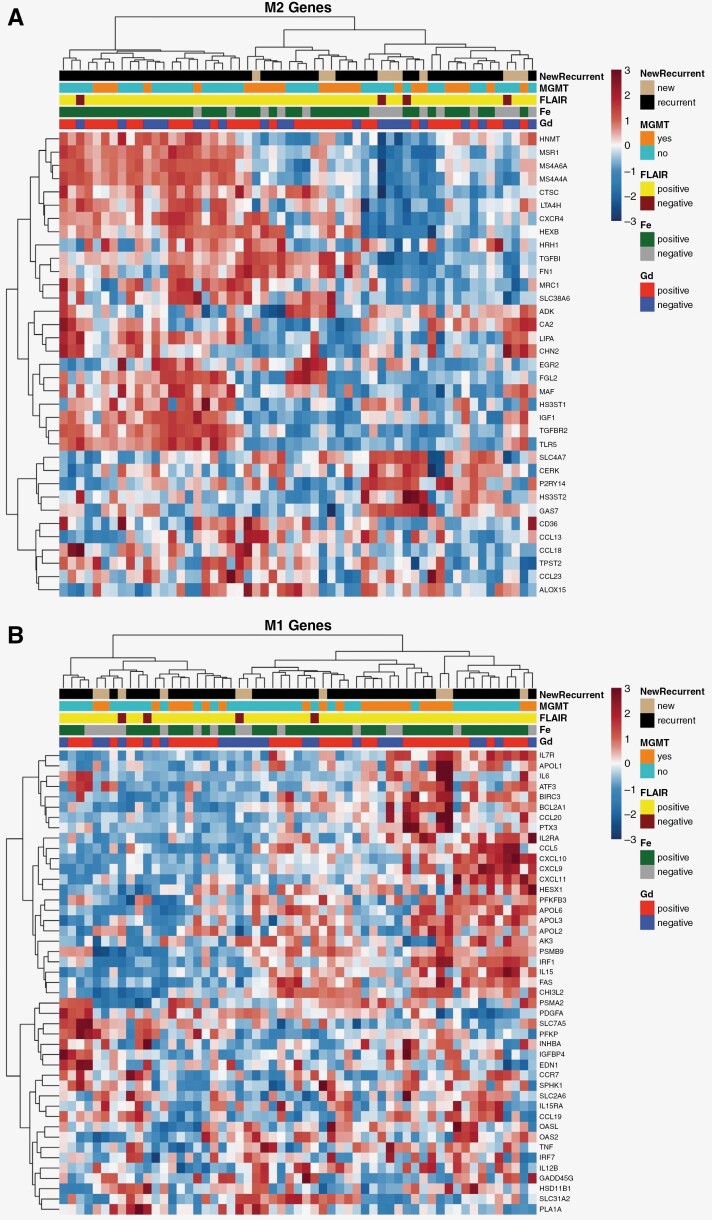
Heatmaps for M1 and M2 macrophage polarization by MRI phenotype.^3,18^ Each column represents a sample taken at the time of biopsy. The corresponding phenotypes and information about the sample are directly above each column. In Yunna et al., the genes associated with M1/M2 macrophage polarization are listed and split into those that are associated with either M1 or M2 macrophage polarization. By splitting the gene set up into M1 or M2 macrophage polarization and displaying the results on separate heatmaps, the contributions of imaging phenotypes become more evident. The leftmost cluster in the M2 heatmap (A), shows that genes are elevated and clustered with Fe+ tissue, suggesting that Fe+ tissues may constitute M2 macrophage polarization. This display of M2 gene upregulation with Fe+ clustering is consistent with the findings in Cibersortx which showed a statistically significant increase in M2 macrophage polarizing in Fe+ tissues. additionally, the leftmost cluster was unique to patients with disease recurrence that was not observed with Gd enhancement alone. The rightmost cluster in the M1 heatmap (B) shows that some genes are elevated and clustered with Fe+ tissue, suggesting that Fe+ tissues may constitute M1 macrophage polarization. However, clustering between M1 macrophage polarization and Fe+ tissue is not as pronounced as the clustering seen in the M2 macrophage heatmaps. Neither heatmap displayed clustering trends with Gd+/Gd– tissue to suggest that either experience elevation or depression in tissue that is either Gd+ or Gd–

To further define the tumor immune microenvironment by Fe-enhancing feature we performed unsupervised hierarchical clustering and visualization through heatmaps of differentially expressed immune processes. Both M2 TAMs (Figure 4) and apoptosis (Supplemental Figure 5) were found to be increased in expression and clustered with the presence of Fe enhancement. Interestingly, a distinct subset of M2 gene expression is suggested by the presence of Fe enhancement within patients with resistance to Stupp Protocol CRT. This is observed by a distinct cluster of M2 gene set enrichment in patients with recurrent disease and Fe enhancement (Figure 4A, leftmost cluster). Fe enhancement was found to cluster with a subset of M1 TAM-related genes; however, clustering was not as pronounced as the clustering seen in the M2 macrophage and apoptosis heatmaps.^19^ None of the heatmaps showed any indication that the presence or absence of Gd enhancement experienced any clustering with upregulated or depressed expression of genes.

## Discussion

In this prospective study, we investigated whether MRI could identify spatially distinct information about the glioblastoma tumor immune microenvironment. Our results suggest all 3 MRI imaging parameters (Gd, Fe, and FLAIR) define overlapping immune profiles. A multiparametric MRI approach to defining tissue characteristics, provided by imaging phenotypes within FLAIR hyperintense regions (Gd+ Fe– or Gd– Fe+), further refined the immune signatures and cellular populations observed with each contrast agent; taking into account spatial differences in contrast enhancement. We observed tissues with a Gd enhancement phenotype (Gd+ Fe–) did not demonstrate any differential immune signature expression. Conversely, the Fe enhancement phenotype (Gd– Fe+) demonstrated marked differential immune signature expression; suggesting the presence of Fe enhancement may account for the observed immune expression profiles in tissue with mixed enhancement phenotype (Gd+ Fe+). Taken together, these findings suggest that the Fe enhancement provides spatially distinct information about innate immune cellular populations within the tumor immune microenvironment that may be distinct at the time of resistance to Stupp protocol CRT. If prospectively validated, the use of multimodal MRI phenotypes has the immediate potential to impact treatment response assessment, timing of therapy, and method of disease monitoring. Furthermore, this minimally invasive imaging methodology could potentially improve upon standard of care Gd-MRI-based response assessment criteria and provide improved diagnostic certainty for patients confronting a currently uncurable disease.

The heterogeneity of the glioblastoma immune microenvironment makes it difficult for physicians to determine prognosis and for treatments to be prescribed with a predictable outcome. Even with CRT, the median survival is only 14.6 months. A better understanding of the inhomogeneities of the immune microenvironment may provide insight into different treatment options as well as allow physicians to monitor treatment response and determine prognosis more accurately. In order to delineate regions of inhomogeneity, stereotactic biopsies were performed and matched with multiparametric MRI phenotypes, utilizing different contrast agents and imaging sequences. Each sample had phenotypes of Gd+/Gd–, Fe+/Fe–, and FLAIR+/FLAIR–. By distinguishing tissue through these phenotypes, we computationally determined immune differences throughout the tumor. Through GSEA, Cibersortx, and R we demonstrated that stratifying tissue by contrast enhancement/FLAIR hyperintensity vs. contrast nonenhancement/FLAIR isointensity was able to detect differences in the tumor immune microenvironment, specifically the immune mechanisms present and immune cellular distribution. These observations show the utility of Fe in describing the neuroinflammatory process which has clinical utility in understanding glioblastoma.

The findings from the initial GSEA computation displayed the potential prognostic capabilities that Gd, FLAIR sequences, and Fe have using Imaging genomics. In the side-by-side comparison of hallmark gene set correlation, both FLAIR and Fe have stronger correlations with gene sets under the process category of immunity when compared to Gd. Further stratification of tissue samples to only include tumor tissue delineated from the FLAIR sequence to evaluate both contrast agents revealed that pairing Gd contrast with a FLAIR sequence has similar capabilities to Fe on its own and paired with a FLAIR sequence. With further stratification of tissue that is FLAIR+ and either Gd+ and Fe– or Gd– and Fe+ showed that immune gene sets express a higher correlation with Fe than with Gd. This suggests that Fe is a better indicator of the condition of the tumor immune microenvironment especially in displaying cell-specific neuroinflammation. Importantly, we build upon previously published data suggesting that Gd-enhancing features delineate glioblastoma immune processes. Our current study findings are consistent with this observation. However, our multiparametric approach with combined Gd and Fe contrast agents may actually suggest that Fe enhancement is the specific imaging feature capable of defining glioblastoma-related immune processes.

The cytoscape results showed that each MRI phenotype has the potential to be a minimally invasive method of understanding the tumor immune microenvironment of glioblastoma. The full utility of the ontology gene set analysis visualized through Cytoscape has not been fully fleshed out. Further analysis will go into how to evaluate the correlation results quantitively. The qualitative representation is sufficient to warrant further investigation.

Cibersortx computed the proportions that immune cells make up within each imaging phenotype. Of these results, the breakdown for Fe was the most interesting. Fe-enhancing tissue suggested the presence of significantly more (*P* < .005) M2 polarized macrophages than tissue that did not enhance with Fe. Additionally, tissue that did not enhance with Fe saw a statistically significant increase (*P* < .05) of activated natural killer cells. M2 macrophage polarization is concerned with an anti-inflammatory process, tumor progression, and immunomodulation, and is associated with hypoxic regions, whereas M1 macrophages are pro-inflammatory.^20,21^ M2 polarized macrophages have been previously implicated in tumor progression and resistance to various treatments. Therefore, knowing the presence of M2 macrophages may delineate a patient population who are developing an immune burden contributing to treatment resistance or where immune therapies such as checkpoint inhibitors may be more beneficial.^22^ The mechanisms of Fe deposition and removal coupled with findings from Cibersortx show that Fe contrast is predictive of genomic signatures present in hypoxic regions of glioblastoma. Understanding the pathology of these regions may allow treating physicians to tailor treatment based on the volume of the hypoxic region marked by the Fe-enhancing phenotype or provide a boost of radiation to this specific region in order to more effectively treat radioresistant regions. Activated natural killer cells have become a point of interest in the realm of immunotherapies, with several clinical trials developing therapies centered around these cells.^23,24^ Our findings show that an absence of Fe enhancement is a strong indicator of regions with higher amounts of activated natural killer cells. The Fe+/Fe– regions of the tumor delineated by the FLAIR+ biomarker, can give insight into the potential for success of activated natural killer cell-based immunotherapies. Fe provides the most utility in assessing response to CRT as well as aiding in treatment decision-making.

Heatmaps allow data visualization of *z*-scored gene expression in the context of imaging biomarkers, including Gd, Fe, and FLAIR as well as pathological biomarkers, including MGMT-methylation. While heatmaps generated incorporated all gene samples, genes were selected and grouped into known gene sets. Of note, the heatmaps for M1 and M2 macrophage polarization were constructed. From this gene set, certain genes are known to be upregulated with M1-polarized macrophages and others are known to be upregulated with M2-polarized macrophages.^18^ Inspection of the genes that are upregulated in this leftmost cluster and that enhance with Fe are normally upregulated with M2 macrophage polarization (Figure 4A). This finding is important in understanding the tumor biology of Fe-enhancing regions but also in the fact that it agrees with the finding from the cellular component analysis from Cibersortx. Both showed that Fe enhancement is correlated with M2 macrophage polarization, providing more validation of this finding.^25,26^ Within Fe-enhancing tissues, apoptosis gene sets were found to be associated with immune response in the GSEA analysis. Fe delineated regions with larger amounts of cell-mediated cell death which could be indicative of the tumor cells’ response to treatment. This was further visualized by unsupervised hierarchical clustering of predefined apoptosis gene sets and tissue sample characteristics. A heatmap of apoptosis gene sets demonstrated that Fe enhancement improved upon localization when compared to Gd-MRI features. This suggests that Fe MRI may provide additional insight into unique biological processes occurring within the tumor microenvironment. These findings warrant further exploration in future studies as they may be useful in treatment selection or monitoring prognosis following treatment.

As shown by each stage of analysis, Fe has utility in localizing neuroinflammation in glioblastoma tissue post-CRT. This is highlighted through the higher correlation to Hallmark and Ontology gene sets when compared directly to Gd, the significantly larger amount of M2 polarized macrophages in Fe+ tissue, and the presence of M2 polarized macrophage genes shown in Figure 4. As this study was exploratory by design and featured 7 patients with 62 tissue samples, its intention was to be hypothesis-generating with respect to delineating macrophage phenotype with Fe-enhanced MRI.

## Conclusions

Fe MRI phenotype builds upon standard of care MRI by delineating glioblastoma neuroinflammatory processes. Specifically, Fe but not Gd enhancement within FLAIR hyperintense regions demonstrate elevated immune-related processes and innate immune cellular constituents. This provides further knowledge about the relationship between Fe contrast enhancement and delineation of the glioblastoma immune microenvironment. If prospectively validated, Fe-enhanced MRI may provide an alternative method for delineating immune cellular components that may provide additional information about treatment outcomes.

## Conflict of Interest statement

Neither any authors nor their immediate family have a financial relationship with a commercial organization that may have a direct or indirect interest in this content.

## Supplementary Material

vdad148_suppl_Supplementary_Data

vdad148_suppl_Supplementary_Figure_S1

vdad148_suppl_Supplementary_Figure_S2

vdad148_suppl_Supplementary_Figure_S3

vdad148_suppl_Supplementary_Figure_S4

vdad148_suppl_Supplementary_Figure_S5

vdad148_suppl_Supplementary_Table_S1

vdad148_suppl_Supplementary_Table_S2

vdad148_suppl_Supplementary_Table_S3

## Notes

Neither any authors nor their immediate family has a financial relationship with a commercial organization that may have a direct or indirect interest in this content.
