# Supplementary material for: Deciphering spatially distinct immune microenvironments in glioblastoma using ferumoxytol and gadolinium-enhanced and FLAIR hyperintense MRI phenotypes
Source: Neurooncol Adv. 2023 Nov 8;5(1):vdad148. doi: 10.1093/noajnl/vdad148 (PMC10699850; doi:10.1093/noajnl/vdad148)
Supplement: vdad148_suppl_Supplementary_Data [file vdad148_suppl_supplementary_data.docx]

**SUPPLEMENTAL INFORMATION**

**SUPPLEMENTAL FIGURES**

**Supplemental Figure 1: Genomic Interrelatedness of Gd-Enhancing Tissues.** The interrelatedness of gene expression from Gd-enhancing tissues is visualized as interactions between established molecular networks and biological pathways. Each dot represents a node denoting at least 1 gene set. A line connecting nodes is called an edge and represents relatedness between the two gene sets. Red nodes are gene sets that are positively correlated, and blue nodes are gene sets that are negatively correlated with the Gd-enhancing MRI phenotype. Tissues from Gd-enhancing regions are associated with an immune response with a strong humoral component.

**Supplemental Figure 2:** **Genomic Interrelatedness of FLAIR Hyperintense Tissues.** The interrelatedness of gene expression from FLAIR hyperintense tissues is visualized as interactions between established molecular networks and biological pathways. Each dot represents a node denoting at least 1 gene set. A line connecting nodes is called an edge and represents relatedness between the two gene sets. Red nodes are positively correlated gene sets, and blue nodes are negatively correlated with the FLAIR hyperintense MRI phenotype. Tissues from FLAIR hyperintense regions are associated with both positive and negative immune regulation.

**Supplemental Figure 3: Cellular Composition of Gd MRI Phenotype.** A) Cibersortx Analysis provides tissue immune cellular composition stratified by Gd MRI phenotype. B) Gd+ tissues were found to have significantly increased levels of neutrophils compared to Gd- tissues. Note: **P<0.005.

**Supplemental Figure 4:** **Cellular Composition of FLAIR MRI Phenotype.** A) Cibersortx Analysis provides tissue immune cellular composition stratified by FLAIR MRI phenotype. B) FLAIR hyperintense tissues were found to have significantly increased levels of neutrophils, eosinophils, M1 macrophages, and resting CD4+ memory T-cells compared to FLAIR isointense tissues. Conversely, FLAIR isointense tissues demonstrated elevated activated CD4+ memory T-cells. Note: *P<0.05, **P<0.005, ***P<0.0005.

**Supplemental Figure 5: Heatmap for Apoptosis Genes.**^18^ Each column represents a single tissue sample taken at the time of biopsy. The corresponding MRI phenotypes, molecular markers, and clinical information are listed above the columns. The genes listed on the right side of this diagram are genes known to be associated with apoptosis; cell-mediated self-destruction. As in the GSEA analysis, this heatmap visualization depicts that with unsupervised clustering, Fe-enhancing (green) and Fe-non-enhancing (grey) samples over and under express different subsets apoptosis-associated genes. A subset of these clustered apoptotic-associated transcripts (top right) is defined by all three Fe+, Gd+, and FLAIR+ MRI features. However, the up regulation of apoptosis gene signatures is best defined by Fe-enhancement.
