# Supplementary figures and images for: Deciphering spatially distinct immune microenvironments in glioblastoma using ferumoxytol and gadolinium-enhanced and FLAIR hyperintense MRI phenotypes
Source: Neurooncol Adv. 2023 Nov 8;5(1):vdad148. doi: 10.1093/noajnl/vdad148 (PMC10699850; doi:10.1093/noajnl/vdad148)

## Slide 1
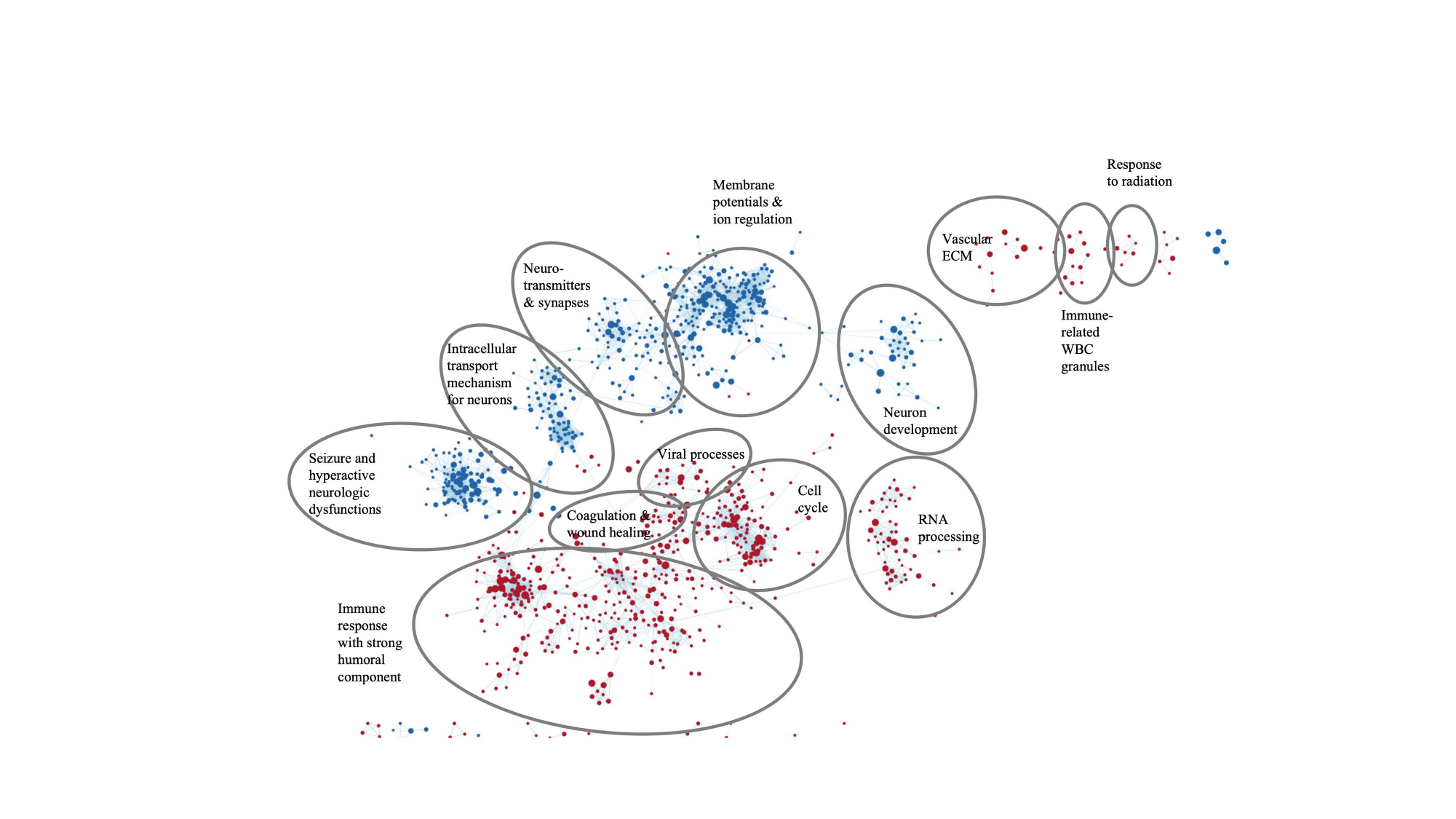

Supplement: vdad148_suppl_Supplementary_Figure_S1 [file vdad148_suppl_supplementary_figure_s1.pptx]

## Slide 1
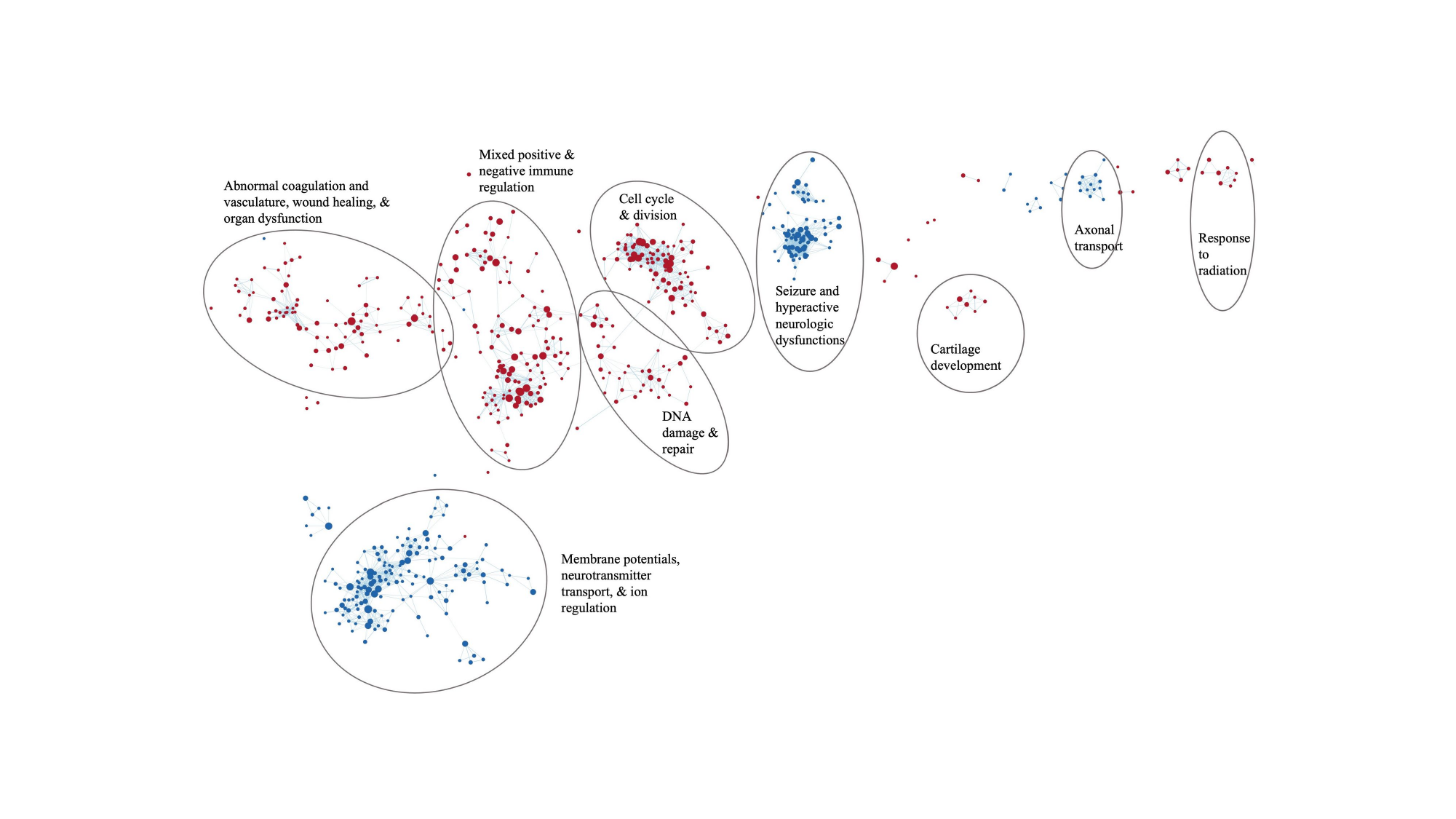

Supplement: vdad148_suppl_Supplementary_Figure_S2 [file vdad148_suppl_supplementary_figure_s2.pptx]

## Slide 1
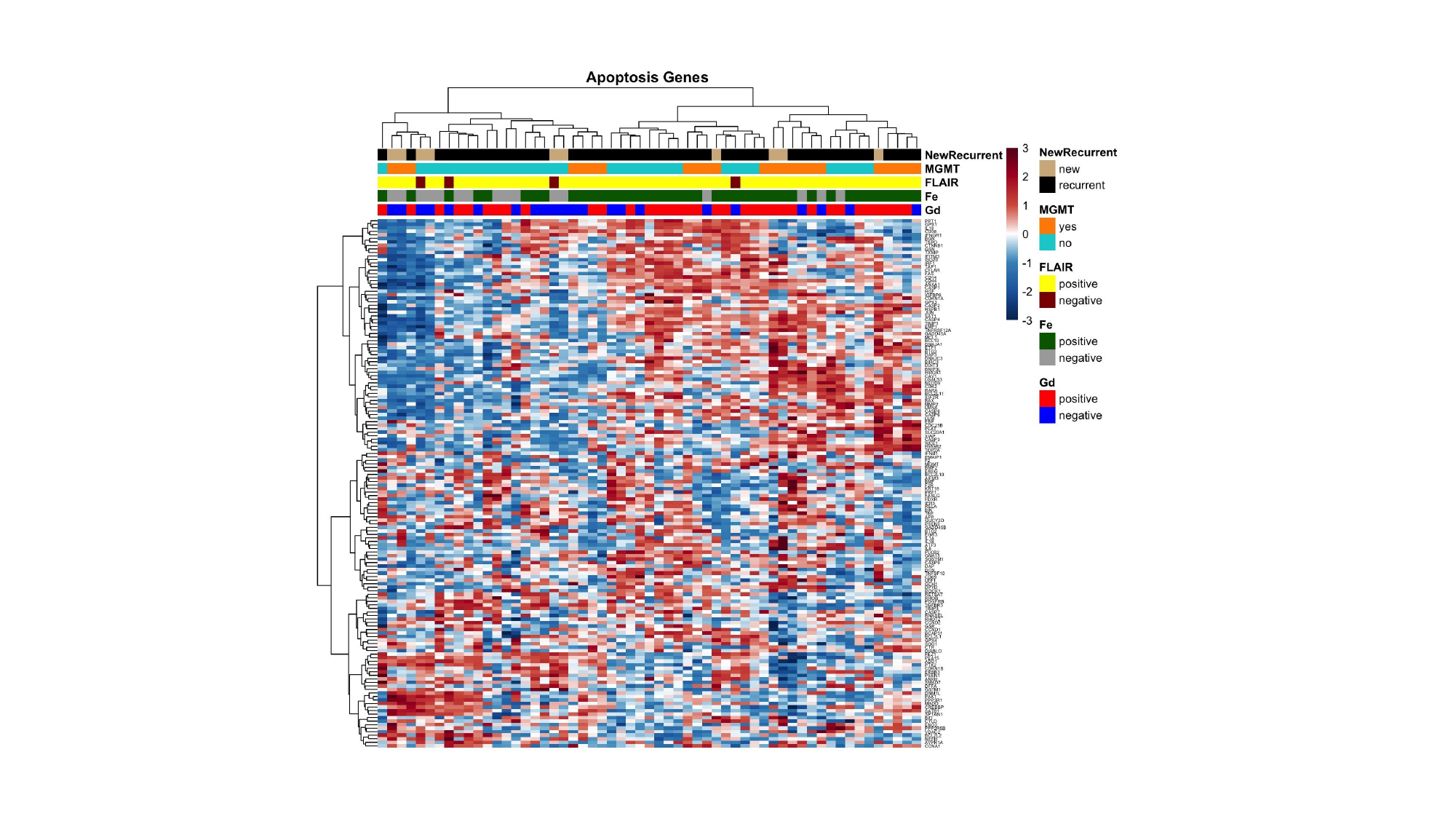

Supplement: vdad148_suppl_Supplementary_Figure_S5 [file vdad148_suppl_supplementary_figure_s5.pptx]
