# Supplementary material for: Deciphering spatially distinct immune microenvironments in glioblastoma using ferumoxytol and gadolinium-enhanced and FLAIR hyperintense MRI phenotypes
Source: Neurooncol Adv. 2023 Nov 8;5(1):vdad148. doi: 10.1093/noajnl/vdad148 (PMC10699850; doi:10.1093/noajnl/vdad148)
Supplement: vdad148_suppl_Supplementary_Table_S1 [file vdad148_suppl_supplementary_table_s1.docx]

| **Supplemental Table 1: Patient Demographic and Tissue Sample Information** | | | | | | | | | | |
| --- | --- | --- | --- | --- | --- | --- | --- | --- | --- | --- |
| **Patient** | **Diagnosis** | **Recurrent** | **IDH Mutant** | **MGMT** | **Age** | **Sex** | **Gd-Fe-** | **Gd-Fe+** | **Gd+Fe+** | **Gd+Fe-** |
| 1 | Astro | N | N | Unk | 45 | M | 3 | 1 | 0 | 0 |
| 2 | GBM | N | N | Y | 77 | M | 2 | 0 | 4 | 0 |
| 3 | GBM | Y | N | N | 77 | M | 0 | 5 | 5 | 2 |
| 4** | GBM | Y | N | N | 44 | M | 0 | 3 | 6 | 0 |
| 4** | GBM | Y | N | N | 45 | M | 4 | 0 | 0 | 2 |
| 5 | GBM | N | Y | Y | 39 | F | 0 | 0 | 2 | 0 |
| 6 | GBM | Y | N | N | 67 | M | 4 | 0 | 0 | 0 |
| 7 | GBM | N | N | N | 53 | F | 2 | 1 | 11 | 0 |
| 8 | GBM | Y | N | N | 58 | M | 1 | 1 | 0 | 4 |
| 9 | BCL | N | NA | NA | 73 | M | 0 | 0 | 0 | 2 |
| 10 | GBM | Y | N | Y | 73 | M | 1 | 2 | 2 | 0 |
| **Note:** GBM = Glioblastoma, Astro = Grade 2 Astrocytoma, BCL = B-cell lymphoma Y = Yes, N = No. NA = not applicable, IDH = isocitrate dehydrogenase, MGMT = O^6^-methylguanine (O^6^-MeG)-DNA methyltransferase methylation status, M = male, F = female. Unk= unkown. *Excluded from analysis due to enrollment criteria. Tissue sample MRI phenotype; Gd+ = T1 weighted Gd enhancement, Gd- = absence of T1 weighted Gd enhancement, Fe+ = T1 weighted Fe enhancement, Fe- = absence of T1 weighted Fe enhancement, FLAIR+ = hyperintense, FLAIR- = isointense. **Sampled twice at first and second recurrence. | | | | | | | | | | |
