# Supplementary material for: Deciphering spatially distinct immune microenvironments in glioblastoma using ferumoxytol and gadolinium-enhanced and FLAIR hyperintense MRI phenotypes
Source: Neurooncol Adv. 2023 Nov 8;5(1):vdad148. doi: 10.1093/noajnl/vdad148 (PMC10699850; doi:10.1093/noajnl/vdad148)
Supplement: vdad148_suppl_Supplementary_Table_S2 [file vdad148_suppl_supplementary_table_s2.docx]

| **Supplemental Table 2: All Differentially Expressed Hallmark Gene Sets by MRI Phenotype** | | |
| --- | --- | --- |
| **Gd+ vs. Gd-** | **FLAIR+ vs. FLAIR-** | **Fe+ vs. Fe-** |
| cell cycle progression: E2F targets | epithelial mesenchymal transition | interferon gamma response |
| cell cycle progression: G2/M checkpoint | cell cycle progression: E2F targets | interferon alpha response |
| epithelial mesenchymal transition | interferon gamma response | cell cycle progression: E2F targets |
| response to hypoxia; HIF1A targets | cell cycle progression: G2/M checkpoint | allograft rejection |
| TNFA signaling via NFκB | TNFA signaling via NFκB | IL6 STAT3 signaling during acute phase response |
| interferon gamma response | interferon alpha response | inflammation |
| MYC targets, variant 1 | response to hypoxia; HIF1A targets | cell cycle progression: G2/M checkpoint |
| mTORC1 signaling | IL6 STAT3 signaling during acute phase response | complement cascade |
| glycolysis and gluconeogenesis | inflammation | epithelial mesenchymal transition |
| interferon alpha response | allograft rejection | blood coagulation cascade |
| IL6 STAT3 signaling during acute phase response | programmed cell death; caspase pathway | TNFA signaling via NFκB |
| blood vessel formation | blood coagulation cascade | IL2 STAT5 signaling |
| blood coagulation cascade | glycolysis and gluconeogenesis | programmed cell death; caspase pathway |
| inflammation |  | KRAS signaling, upregulated genes |
| unfolded protein response; ER stress |  | response to hypoxia; HIF1A targets |
| complement cascade |  | blood vessel formation |
| programmed cell death; caspase pathway |  |  |
| allograft rejection |  |  |
| MYC targets, variant 2 |  |  |
| **Note:** Gene set enrichment analysis was carried out by GSEA with Hallmark Gene Sets. This table shows the hallmark genes sets that were upregulated with Gd+ vs. Gd-, FLAIR+ vs. FLAIR-, and Fe+ vs. Fe- in decreasing NES. Gd+ = T1 weighted Gd enhancement, Gd- = absence of T1 weighted Gd enhancement, Fe+ = T1 weighted Fe enhancement, Fe- = absence of T1 weighted Fe enhancement, FLAIR+ = hyperintense, FLAIR- = isointense. | | |
