# Supplementary material for: Deciphering spatially distinct immune microenvironments in glioblastoma using ferumoxytol and gadolinium-enhanced and FLAIR hyperintense MRI phenotypes
Source: Neurooncol Adv. 2023 Nov 8;5(1):vdad148. doi: 10.1093/noajnl/vdad148 (PMC10699850; doi:10.1093/noajnl/vdad148)
Supplement: vdad148_suppl_Supplementary_Table_S3 [file vdad148_suppl_supplementary_table_s3.docx]

| **Supplemental Table 3: Description of M1/M2 Polarized Macrophage Genes**^25^ | | |
| --- | --- | --- |
| **Gene** | **Full Name** | **Description** |
| **M1** | | |
| *CCR7* | C-C motif chemokine receptor 7 | Encodes a protein that influences T-cell migration to inflamed regions |
| *IL2RA* | interleukin 2 receptor subunit alpha | Encodes for an integral transmembrane protein |
| *IL15RA* | interleukin 15 receptor subunit alpha | Encodes for a protein that increases cell proliferation and inhibits apoptosis |
| *IL7R* | interleukin 7 receptor | Encodes for a protein that is involved in lymphocyte V(D)J recombination |
| *CXCL11* | C-X-C motif chemokine ligand 11 | Encodes for a protein that is involved in immune processes |
| *CCL19* | C-C motif chemokine ligand 19 | Encodes for a protein involved in inflammation and immune system regulation |
| *CXCL10* | C-X-C motif chemokine ligand 10 | Encodes for a protein that is involved in immune cell stimulation |
| *CXCL9* | C-X-C motif chemokine ligand 9 | Encodes for a protein involved in inflammation and immune system regulation |
| *TNF* | tumor necrosis factor | Encodes for a protein that is a proinflammatory cytokine |
| *CCL5* | C-C motif chemokine ligand 5 | Encodes for a protein involved in inflammation and immune system regulation |
| *CCL15* | C-C motif chemokine ligand 15 | Encodes for a protein that is involved in immune cell stimulation |
| *IL12B* | interleukin 12B | Encodes for a protein that is involved in immune cell stimulation |
| *IL15* | interleukin 15 | Encodes for a protein that is involved in immune cell stimulation |
| *TRAIL* | TNF superfamily member 10 | Encodes for a protein that is a proinflammatory cytokine |
| *IL6* | interleukin 6 | Encodes for a cytokine involved in B cell maturation and inflammation |
| *CCL20* | C-C motif chemokine ligand 20 | Encodes for a protein that is a proinflammatory cytokine |
| *PBEF1* | nicotinamide phosphoribosyltransferase | Encodes for a protein that is involved in metabolic activity |
| *ECGF1* | sphingosine-1-phosphate receptor 1 | Encodes for a protein that is involved with G-coupled protein receptors |
| *BCL2A1* | BCL2 related protein A1 | Encodes for a protein that may be involved with tumorigenesis |
| *FAS* | Fas cell surface death receptor | Encodes for a protein involved in apoptosis regulation |
| *BIRC3* | baculoviral IAP repeat containing 3 | Encodes for a protein involved in apoptosis inhibitor |
| *GADD45G* | growth arrest and DNA damage inducible gamma | This gene is expressed more in conditions of stressful growth arrest |
| *HSXIAPAF1* | XIAP associated factor 1 | Encodes for a protein involved in inflammatory regulation |
| *SLC7A5* | solute carrier family 7 member 5 | Encodes for a protein involved in transmembrane transport |
| *SLC21A15* | solute carrier organic anion transporter family member 5A1 | Encodes for a protein involved in transmembrane transport |
| *SLC2A6* | solute carrier family 2 member 6 | Encodes for a protein involved in transmembrane transport |
| *SLC31A2* | solute carrier family 31 member 2 | Encodes for a protein involved in transmembrane transport |
| *INDO* | indoleamine 2,3-dioxygenase 1 | Encodes for a protein involved in antitumoral processes |
| *PLA1A* | phospholipase A1 member A | Encodes for a protein involved in inflammation and immune system regulation |
| *OASL* | 2'-5'-oligoadenylate synthetase like | Encodes for a protein involved DNA binding |
| *CHI3L2* | chitinase 3 like 2 | Encodes for a protein involved in generating cartilage |
| *HSD11B1* | hydroxysteroid 11-beta dehydrogenase 1 | Encodes for a protein that acts as a catalyst in cortisol conversion |
| *AK3* | adenylate kinase 3 | Encodes for a phosphotransferase |
| *SPHK1* | sphingosine kinase 1 | Encodes for a phosphotransferase |
| *PFKFB3* | 6-phosphofructo-2-kinase/fructose-2,6-biphosphatase 3 | Encodes for a kinase |
| *PSME2* | proteasome activator subunit 2 | Encodes for a immunoproteasome |
| *PFKP* | phosphofructokinase, platelet | Encodes for a protein involved in reprogramming of metabolic activity |
| *PSMB9* | proteasome 20S subunit beta 9 | Encodes for a immunoproteasome |
| *PSMA2* | proteasome 20S subunit alpha 2 | Encodes for a immunoproteasome |
| *OAS2* | 2'-5'-oligoadenylate synthetase 2 | Encodes for a protein involved in nonspecific immunity |
| *PTX3* | pentraxin 3 | Encodes for a protein involved in inflammation |
| *CSPG2* | versican | Encodes for a protein involved in proliferation |
| *APOL3* | apolipoprotein L3 | Encodes for a protein involved in lipid and cholesterol carriage |
| *IGFBP4* | insulin like growth factor binding protein 4 | Encodes for a protein involved binding with insulin-like growth factor |
| *APOL1* | apolipoprotein L1 | Encodes for a protein involved in lipid and cholesterol carriage |
| *PDGFA* | platelet derived growth factor subunit A | Encodes for a protein involved in lipid and cholesterol carriage |
| *EDN1* | endothelin 1 | Encodes for a protein involved made up of PDGF and VGEF |
| *APOL2* | apolipoprotein L2 | Encodes for a protein involved in lipid and cholesterol carriage |
| *INHBA* | inhibin subunit beta A | Encodes for a protein involved in hormone secretion in the pituitary gland |
| *APOL6* | apolipoprotein L6 | Encodes for a protein involved in lipid and cholesterol carriage |
| *HESX1* | HESX homeobox 1 | Encodes for a protein involved in developmental regulation |
| *IRF1* | interferon regulatory factor 1 | Encodes for a protein that is a tumor suppressor |
| *ATF3* | activating transcription factor 3 | Encodes for a protein that is involved in cancer cell signaling |
| *IRF7* | interferon regulatory factor 7 | Encodes for a protein that is involved in the immune response |
| **M2** | | |
| *GPR86* | purinergic receptor P2Y13 | Encodes for a protein that is involved with G-coupled protein receptors |
| *P2RY5* | lysophosphatidic acid receptor 6 | Encodes for a protein that is involved with G-coupled protein receptors |
| *TGFBR2* | transforming growth factor beta receptor 2 | Encodes for a protein that is involved in immunosuppression and tumorigenesis |
| *HRH1* | histamine receptor H1 | Encodes for a protein that is involved with G-coupled protein receptors |
| *TLR5* | toll like receptor 5 | Encodes for a protein that is involved with pathogen recognition as a toll-like receptor |
| *DCL-1* | CD302 molecule | Encodes for a receptor involved in phagocytosis |
| *MSR1* | macrophage scavenger receptor 1 | Encodes for a receptor involved in phagocytosis |
| *CXCR4* | C-X-C motif chemokine receptor 4 | Encodes for a protein that is a CXC chemokine receptor |
| *DECTIN1* | C-type lectin domain containing 7A | Encodes for a membrane protein |
| *P2RY14* | purinergic receptor P2Y14 | Encodes for a protein that is involved with G-coupled protein receptors |
| *DCSIGN* | CD209 molecule | Encodes for a protein involved in pathogen recognition |
| *CLECSF13* | C-type lectin domain containing 10A | Encodes for a protein involved in inflammation and immune response |
| *MS4A6A* | membrane spanning 4-domains A6A | Encodes for a protein involved in cell cycle control |
| *CD36* | CD36 molecule | Encodes for a protein involved in cell adhesion |
| *MS4A4A* | membrane spanning 4-domains A4A | Encodes for a protein involved in cell cycle control |
| *MRC1* | mannose receptor C-type 1 | Encodes for a protein that assists macrophages in endocytosis |
| *IGF1* | insulin like growth factor 1 | Encodes for a protein that is an insulin analogue |
| *CCL23* | C-C motif chemokine ligand 23 | Encodes for a protein involved in inflammation |
| *CCL18* | C-C motif chemokine ligand 18 | Encodes for a protein involved in inflammation |
| *CCL13* | C-C motif chemokine ligand 13 | Encodes for a protein involved in inflammation |
| *SLC21A9* | solute carrier organic anion transporter family member 2B1 | Encodes for a protein involved in transmembrane transport |
| *SLC4A7* | solute carrier family 4 member 7 | Encodes for a protein involved in transmembrane transport |
| *SLC38A6* | solute carrier family 38 member 6 | Encodes for a protein involved in transmembrane transport |
| *CTSC* | cathepsin C | Encodes for a protein involved in proteolysis for the immune system |
| *HEXB* | hexosaminidase subunit beta | Encodes for a protein involved in the G2M checkpoint |
| *LIPA* | lipase A, lysosomal acid type | Encodes the protein lipase A |
| *ADK* | adenosine kinas | Encodes for adenosine kinase |
| *HNMT* | histamine N-methyltransferase | Encodes for one of the enzymes that metabolizes histamine |
| *TPST2* | tyrosylprotein sulfotransferase 2 | Encodes an integral membrane protein |
| *CERK* | ceramide kinase | Encodes for a protein involved in apoptosis, inflammation, and phagocytosis |
| *HS3ST2* | heparan sulfate-glucosamine 3-sulfotransferase 2 | Encodes an integral membrane protein expressed primarily in the brain |
| *LTA4H* | leukotriene A4 hydrolase | Encodes a protein that acts as a proinflammatory mediator |
| *CA2* | carbonic anhydrase 2 | Encodes a carbonic anhydrase enzyme |
| *ALOX15* | arachidonate 15-lipoxygenase | Encodes a protein that regulates inflammation and immunity |
| *HS3ST1* | heparan sulfate-glucosamine 3-sulfotransferase 1 | Encodes a protein involved in anticoagulation |
| *TGFBI* | transforming growth factor beta induced | Encodes for a protein involved in cell adhesion |
| *SEPP1* | selenoprotein | Encodes for a protein that acts as an extracellular antioxidant |
| *CHN2* | chimerin 2 | Encodes a protein involved in the structure of Schwann cells |
| *FN1* | fibronectin 1 | Encodes for a protein involved in cell adhesion |
| *FGL2* | fibrinogen like 2 | Encodes a protein that has functions in mucosal regions |
| *GAS7* | growth arrest specific 7 | Encodes a protein involved in neuronal development |
| *EGR2* | early growth response 2 | Encodes a protein that is a transcription factor |
| *MAF* | MAF bZIP transcription factor | Encodes a protein that moderates transcription |
